# Phrenic nerve palsy and pulsed field ablation procedures for atrial fibrillation

**DOI:** 10.1093/europace/euae202

**Published:** 2024-08-09

**Authors:** Frederic Franceschi, Linda Koutbi, Baptiste Maille

**Affiliations:** Department of Cardiology, CHU Timone, 264 Rue Saint Pierre, 13005 Marseille, France; Department of Cardiology, Aix-Marseille University, 58 Boulevard Charles Livon, 13284 Marseille, France; Center for CardioVascular and Nutrition Research (C2VN), INSERM, INRA, 27 Boulevard Jean Moulin, 13005 Marseille, France; Department of Cardiology, CHU Timone, 264 Rue Saint Pierre, 13005 Marseille, France; Department of Cardiology, Aix-Marseille University, 58 Boulevard Charles Livon, 13284 Marseille, France; Department of Cardiology, CHU Timone, 264 Rue Saint Pierre, 13005 Marseille, France; Department of Cardiology, Aix-Marseille University, 58 Boulevard Charles Livon, 13284 Marseille, France; Center for CardioVascular and Nutrition Research (C2VN), INSERM, INRA, 27 Boulevard Jean Moulin, 13005 Marseille, France

**Keywords:** Pulsed field ablation, Phrenic nerve palsy, Compound motor action potential, Farapulse, Atrial fibrillation, Ablation, Complication

We read with great interest the study by Ollitrault *et al*.^1^ Beyond the small sample size, it seems to present several limitations concerning the occurrence of phrenic nerve palsy (PNP).

Firstly, phrenic nerve (PN) stimulation is performed using a pentaspline pulsed field ablation (PFA) catheter. The method is not detailed (stimulation level, amplitude and pulse width, etc.). This is an unusual approach, as this therapeutic catheter is not designed for stimulation. The risk, as highlighted in our recently published case report,^2^ is to stimulate the nerve below the level of conduction block and thus underestimate the incidence of PNP.

Secondly, no compound motor action potential (CMAP) values were reported in the study. The authors defined PN stunning as a drop in CMAP amplitude > 30% after application of PFA. This threshold is arbitrary. Incomplete CMAP recovery is probably not trivial, as CMAP amplitude reflects the number of diaphragmatic fibres that depolarize. For example, is the fact that 25% of fibres no longer depolarize after PFA a fact that should not be taken into account? What are the long-term consequences? To date, no one can answer these questions.

Thirdly, chest X-rays were performed in only 20 patients. This figure is far too low. We also know that chest X-rays can be falsely reassuring^3^ and that dynamic tests such as ultrasound or fluoroscopic sniff test should be preferred.

In addition, a case of delayed permanent PNP has already been reported with PFA.^4^ Several cases of delayed diaphragmatic paralysis have been reported in the literature, outside the field of atrial fibrillation (AF) ablation.^5,6^ The mechanism remains unclear, but as patients with PNP are asymptomatic in 90% of cases, delayed forms may be difficult to diagnose. Relying solely on what happens at the time of the procedure could be falsely reassuring.

Finally, pentaspline applications in the superior vena cava are parallel to the PN, unlike applications in the right pulmonary veins, which are perpendicular to the nerve. Assuming a traumatic mechanism, perpendicular applications should be more deleterious.

Under these conditions, no conclusion can be drawn from this study^1^ as to the incidence of PNP in pulmonary veins isolation with PFA.

Nothing is known about the intimate phenomenon that leads to PNP during PFA procedures; nevertheless, several mechanisms could be involved. The first is probably purely electrical and could explain the transient phenomena. The second could be traumatic, with axonal degeneration below the level of trauma, and explain the persistent phenomena. Phrenic nerve palsy is a complication of AF ablation procedures,^7^ and it is undoubtedly also a complication of PFA. Its incidence was 1.3% in the ADVENT study,^8,9^ where 3 patients (1%) recovered only at the end of the follow-up at 1 year. This incidence has been reported in the absence of specific PN monitoring. On the other hand, in the MANIFEST 17K study,^10^ no persistent PNP was reported. However, the quality of data from this type of retrospective registry is probably much lower than that of a randomized trial such as ADVENT. Other studies have reported similar reassuring results regarding the incidence of diaphragmatic paralysis.^11-14^ All these data have the same limitations: these are mainly retrospective observational studies where diaphragmatic monitoring is never performed. The true incidence of PNP in PFA procedures is therefore unknown, as systematic diaphragm monitoring has never been performed in a large-scale cohort study.

We believe it is imperative to remain vigilant and plan prospective trials targeted to appropriately detect persistent PNP, in order to assess its precise occurrence as a complication of PFA.
